# Cyclic GMP signaling and mitochondrial BK channels in cardioprotection against ischemia/reperfusion injury

**DOI:** 10.1186/2050-6511-16-S1-A50

**Published:** 2015-09-02

**Authors:** Sandra Frankenreiter, Angelina Kniess, Peter Ruth, Thomas Krieg, Andreas Friebe, Robert Lukowski

**Affiliations:** 1 Department of Pharmacology, Toxicology and Clinical Pharmacy, Institute of Pharmacy, University of Tübingen, Tübingen, Germany; 2 Department of Medicine, University of Cambridge, Cambridge, United Kingdom; 3 Physiologisches Institut, Julius-Maximilians Universität, Würzburg, Germany

## Background

By studying hearts obtained from global BK-null mice (BK-KO) in an ex vivo Langendorff perfusion setup we and others previously found evidence for mitochondrial BK channels (mitoBKs) in cardiomyocytes as infarct-limiting factors [1,2]. It is well established that canonical BK channels usually present at the plasma membrane of cells are directly stimulated by the cyclic guanosine-3',5'-monophosphate (cGMP)/cGMP-dependent protein kinase type I pathway; however, it is unclear whether cardioprotection afforded by cardiomyocyte (CM) cGMP in vivo requires mitoBK.

## Methods

Using the Cre/loxP recombination system we generated animals with a CM-restricted deletion of BK channels (CMBK-KO) [3] and of nitric oxide (NO)-sensitive guanylyl cyclases (CMsGC-KO) [4]. The susceptibility of the conditional mutants to ischemia/reperfusion (I/R) injury was compared to age- and litter-matched controls (CMBK-CTR and CMsGC-CTR) as well as to global BK-KO and BK wild-type (BK-WT) mice. An open chest in situ model of myocardial infarction was applied to determine differences in infarct size at baseline and upon ischemic pre-/postconditioning (iPre/iPost) or pharmacological interventions using either the BK blocker paxilline, the BK opener NS11021 or the phosphodiesterase 5 (PDE5) inhibitors sildenafil and tadalafil, which are cardioprotective agents that should signal via increasing cGMP [5,6].

## Results

Baseline infarct size of CMsGC-KOs did not differ from that of CMsGC-CTRs, however, 30 min of ischemia followed by 120 min of reperfusion provoked significantly more cardiac damage in global BK-KO and CMBK-KO mice than in age- and litter-matched BK-WTs and CMBK-CTRs, respectively.

The BK blocker paxilline (applied 5 min after the onset of ischemia) did not affect the response to I/R of CMBK-KO hearts, whereas in hearts from CMBK-CTR mice we observed an increase in the cardiac damage. With the BK opener NS11021 (5 min before reperfusion) we observed a more drastic decrease in the infarct size in hearts from CMBK-CTR than in CMBK-KO mice.

As expected, short repetitive episodes of ischemia applied directly after infarction (iPost) significantly reduced the myocardial damage in all WT/CTR hearts, whereas protection afforded by iPost was less pronounced in the absence of mitoBK channels and completely abolished in hearts lacking CM NO-sGC.

Interestingly, cardioprotection elicited either by an intra-atrial injection of sildenafil (5 min before reperfusion) or by an i.p. injection of tadalafil (60 min before the ischemic insult) also seems to require mitoBK and NO-GC in the CM.

## Conclusion

In summary, the presented findings demonstrate that the lack of CM BK channels renders the heart more susceptible to I/R injury. Cardioprotection elicited by the BK opener NS11021 suggests that BK channels may be promising drug targets that interfere with the causes and/or consequences of myocardial ischemia. Interestingly, both CM NO-GC and BK are important to allow the protective signaling events triggered either by short, repetitive episodes of ischemia or by agents targeting PDE5. Further studies are needed to elucidate whether cardioprotection via NO-GC and BK are linked by a common cGMP pathway in the CM itself.

## Note

Sandra Frankenreiter, Angelina Kniess, Peter Ruth, Andreas Friebe and Robert Lukowski are members of the DFG Research Unit 2060 “cGMP signalling in cell growth and survival”.
